# Suppression of CaMKIIβ Inhibits ANO1-Mediated Glioblastoma Progression

**DOI:** 10.3390/cells9051079

**Published:** 2020-04-26

**Authors:** Kyoung Mi Sim, Young-Sun Lee, Hee Jin Kim, Chang-Hoon Cho, Gwan-Su Yi, Myung-Jin Park, Eun Mi Hwang, Jae-Yong Park

**Affiliations:** 1 School of Biosystems and Biomedical Sciences, College of Health Sciences, Korea University, Seoul 02841, Korea; yhotff@korea.ac.kr (K.M.S.); ssunny@korea.ac.kr (Y.-S.L.); gmlwls2658@naver.com (H.J.K.); chois007@gmail.com (C.-H.C.); 2 Center for Functional Connectomics, KIST, Seoul 02792, Korea; 3 Division of Radiation Cancer Research, Research Center for Radio-Senescence, Korea Institute of Radiological and Medical Sciences, Seoul 01812, Korea; mjpark@kirams.re.kr; 4 Department of Bio and Brain Engineering, KAIST, Daejeon 34141, Korea; gwansuyi@gmail.com

**Keywords:** ANO1, CaMKIIβ, U251 glioblastoma cells, U87 MG glioblastoma cells

## Abstract

ANO1, a Ca^2+^-activated chloride channel, is highly expressed in glioblastoma cells and its surface expression is involved in their migration and invasion. However, the regulation of ANO1 surface expression in glioblastoma cells is largely unknown. In this study, we found that Ca^2+^/Calmodulin-dependent protein kinase II (CaMKII) β specifically enhances the surface expression and channel activity of ANO1 in U251 glioblastoma cells. When KN-93, a CaMKII inhibitor, was used to treat U251 cells, the surface expression and channel activity of ANO1 were significantly reduced. Only CaMKIIβ, among the four CaMKII isoforms, increased the surface expression and channel activity of ANO1 in a heterologous expression system. Additionally, gene silencing of CaMKIIβ suppressed the surface expression and channel activity of ANO1 in U251 cells. Moreover, gene silencing of CaMKIIβ or ANO1 prominently reduced the migration and invasion of U251 and U87 MG glioblastoma cells. We thus conclude that CaMKIIβ plays a specific role in the surface expression of ANO1 and in the ANO1-mediated tumorigenic properties of glioblastoma cells, such as migration and invasion.

## 1. Introduction

Glioblastoma (astrocytomas, WHO grade IV) is the most common and lethal type of primary brain tumor [1,2]. The mean survival time of patients with glioblastoma is still no more than 12 to 15 months from initial diagnosis despite maximum treatment consisting of chemotherapy, surgery, and radiotherapy [3]. A distinctive feature contributing to the aggressiveness of the disease is the special ability of glioblastoma cells to actively migrate along brain blood vessels [4,5]. Glioblastomas have several ion channels that have a good ability to push out osmotically active small molecules, leading to cell shrinkage due to the release of cytoplasmic water [6]. Channels providing the electrochemical momentum for ion movement in glioblastoma cells are the chloride channel ClC-3 [7], large-conductance voltage- and Ca^2+^-activated potassium (BK) channel [8], and Ca^2+^ permeable alpha-amino-3-hydroxy-5-methyl-4-isoxazolepropionate (AMPA) channels [9]. Recently, we also reported that ANO1 (Anoctamin1 or TMEM16A) is involved in tumorigenesis of glioblastoma cells [10,11].

ANO1 was identified as a Ca^2+^-activated Cl^−^ channel (CaCC) that is activated by intracellular Ca^2+^ [12,13,14] and plays important roles in the physiological functioning of diverse tissues, such as the salivary glands, trachea, pancreas, gut, and mammary glands [15,16,17]. ANO1 also contributes to spontaneous firing in cholinergic neurons of the medial habenula [18]. In addition to normal tissues, ANO1 is highly expressed in various types of cancers, including head and neck squamous cell carcinoma (HNSCC), breast cancer, pancreatic cancer, prostate cancer, thyroid cancer, and glioblastoma [19,20,21,22]. Because of the cancer-related increase in its expression, ANO1 is also known by different names, such as DOG-1 (discovered on gastrointestinal stromal tumors protein 1), ORAOV2 (oral cancer overexpressed 2), and TAOS2 (tumor-amplified and overexpressed sequence 2) [23,24]. Based on the overexpression of ANO1 in diverse cancers, ANO1 may be an important ion channel for cancer development and metastasis.

Several recent studies have shown that ANO1 is involved in oncogenic signaling. In HNSCC, ANO1 contributes to the activation of mitogen-activated protein kinase (MAPK) and protein kinase B (PKB; also known as AKT) by interacting with EGFR [20]. The activation of EGFR signaling by ANO1 also promotes the activation of Ca^2+^/Calmodulin-dependent kinase II (CAMKII) signaling in breast cancer cells [20]. In addition, overexpression of ANO1 activates nuclear factor-κB (NF-κB) signaling, and knockdown of ANO1 suppresses the proliferation, migration, and invasion of glioma cells [22]. We also reported that the surface expression of ANO1 was increased by interaction with 14-3-3γ in several glioblastoma cell lines, and ANO1 surface expression is critical for the migration and invasion of glioblastoma cells [10,11]. Although ANO1 surface expression is important in the tumorigenesis of glioblastoma cells, the detailed molecular mechanisms underlying ANO1-surface expression remain to be fully understood.

The CaMKII family of proteins are multifunctional serine/threonine-specific protein kinases that are regulated by the Ca^2+^/Calmodulin complex [25]. The CaMKII family has important functions in the regulation of the differentiation, proliferation, and survival of various normal cells, as well as cancer cells [26]. CaMKII proteins are encoded by four genes: CAMK2A, CAMK2B, CAMK2G, and CAMK2D, which produce CaMKIIα, CaMKIIβ, CaMKIIγ, and CaMKIIδ, respectively [25]. These CaMKII proteins phosphorylate nearly 40 different proteins, including enzymes, kinases, transcription factors, and ion channels [26,27,28]. Interestingly, recent studies reported that the channel activity of ANO1 is inhibited by CaMKII-dependent phosphorylation. In basilar arterial smooth muscle cells (BASMCs), CaMKII*γ* inhibits native CaCC currents, and the serine 727 mutant (S727A) of ANO1 reverses the CaMKII*γ*-mediated inhibition of ANO1-mediated currents [29]. Another study also reported that ANO1-mediated currents are inhibited by CaMKII in HEK-293 cells and serine 528 of ANO1 is an important contributor to the inhibition of ANO1-mediated currents by CaMKII [30]. Despite the different sites of ANO1 suggested as CaMKII-mediated phosphorylation sites, these studies demonstrated similar results, showing CaMKII-mediated ANO1 inhibition in BASMCs and HEK-293 cells. Although our previous study showed that tumorigenesis of glioblastoma cells was suppressed by inhibition of ANO1 [10], the effect of CaMKII on the activity of ANO1 has never been studied in glioblastoma cells.

In the present study, we found that surface expression and channel activity of ANO1 are enhanced in a CaMKIIβ-specific manner in U251 and U87 MG glioblastoma cells. We also found that suppression of CaMKIIβ using small interfering RNA (siRNA) or a CaMKII inhibitor, KN-93, reduced the surface expression and channel activity of ANO1 in glioblastoma cells. In addition, specific gene silencing of CaMKIIβ and/or ANO1 suppressed the migration and invasion of glioblastoma cells. Our results provide strong evidence for a CaMKIIβ-dependent regulation mechanism of ANO1 surface expression and indicate potential therapeutic targets against ANO1-mediated tumorigenesis in glioblastoma cells.

## 2. Materials and Methods

### 2.1. Chemicals

4,4′-Diisothiocyano-2,2′-stilbenedisulfonic acid (DIDS), a broad-spectrum chloride channel blocker was purchased from Tocris (Minneapolis, MN, USA, 4523). CaCCinh-A01, an ANO1 inhibitor, and KN-93, a CaMKII inhibitor were purchased from Sigma-Aldrich (St.Louis, MO, USA, 422708). 4′,6-diamidino-2-phenylindole (DAPI) staining solutions was purchased from Thermo Fisher Scientific (Waltham, MA, USA, 62248). Other reagents were purchased from Sigma-Aldrich.

### 2.2. Cell Culture and Transfection

U251 and U87 MG glioblastoma cell lines were cultured in Dulbecco’s modified Eagle’s medium (DMEM) (Hyclone, Pittsburgh, PA, USA, SH30243.01) containing 10% fetal bovine serum (FBS) (Gibco, Waltham, MA, USA, 16000-044) and 100 units/mL penicillin-streptomycin (Gibco, 15140122). Cultures were maintained at 37 °C in a humidified, 5% CO_2_ -containing atmosphere. Cells were seeded on poly-L-lysine (10 μg/mL) pre-coated plates and then expression vectors for CaMKII isoforms were transfected into cells using jet PEI (Polyplus, Seoul, Korea, 101-10N) according to the manufacturer’s protocol. To silence CaMKIIβ expression, CaMKIIβ small-interfering RNA (CaMKIIβ siRNA) (Santa Cruz, Dallas, TX, USA, sc-38951) was transfected using INTERFERin^®^ siRNA transfection reagent (Polyplus, 409-10), according to the manufacturer’s protocol.

### 2.3. Production and Infection With Lentivirus

To produce lentivirus containing specific short hairpin-forming RNA against ANO1 (ANO1 shRNA), we purchased the TMEM16 (ANO1) Human shRNA Plasmid Kit from OriGene (Rockville, Maryland, USA, TL300993), which comprises a control lentiviral scrambled shRNA vector (Sc shRNA) and four individual lentiviral vectors encoding ANO1 shRNAs (shANO1A~shANO1D). Among the four lentiviral vectors encoding ANO1 shRNAs, we selected the most efficient lentiviral vector containing shANO1D. Sequences of shANO1D is as follows: 5′-GCTGAATACGAAGCCAGAGTCTTGGAGAA-3′. Using the control lentiviral vector encoding Sc shRNA and lentiviral vectors encoding ANO1 shRNA, lentivirus production was performed according to the protocol described in a previous report [31]. Viral particles were concentrated and purified using Lenti-X concentrator (Clontech, Kyoto, Japan, 631231). U251 and U87 MG cells were seeded and cultured in fibronectin-coated 60 mm plates (5 × 10^5^ cells/dish) for 24 h and then infected with lentivirus in the presence of polybrene (8 µg/mL) for 72 h.

### 2.4. Construction of Expression Vectors

To construct expression vectors, we routinely used the Gateway recombination cloning system (Invitrogen). The BP clones encoding human CaMKIIα (NM_015981.3, Addgene, Watertown, Massachusetts, USA, 23408), human CaMKIIβ (NM_172080.2, Addgene, 23820), human CaMKIIδ (NM_172127.2, Addgene, 23814), and human CaMKIIγ (NM_172170.5, Addgene, 23409) were purchased from Addgene. These genes were transferred from BP clones to several destination expression vectors, such as pDEST-HA-N, pDEST-Flag-N, and pDEST-mCherry-N by the Gateway LR recombination reaction (Invitrogen), according to the manufacturer’s guidelines.

### 2.5. Reverse Transcription-Polymerase Chain Reaction (RT-PCR) and Quantittative Real Time PCR (qPCR)

Total RNA was isolated from U251 cells or U87 MG cells using an RNA purification Kit (GeneAll, Seoul, Korea, 305-101), according to the manufacturer’s instructions. RT was performed with 1 μg total RNA using a cDNA Synthesis Kit (Biofact, Daejeon, Korea, BR546-096), according to the manufacturer’s instructions. PCR was performed using Pfu Plus 5x PCR premix (Elpis, seoul, Korea, EBT-1405) under the following cycle conditions: Denaturation at 95 °C for 20 s, annealing at 55 °C for 20 s, and extension at 72 °C for 30 s. This cycle was repeated a total of 30 times. The PCR products were separated by electrophoresis in a 2% agarose gel, and images were captured on a gel imaging system. qPCR was also performed with SYBR Green mix (Enzo, New York, USA, ENZ-NUC104-1000). Table 1 shows primer sets for CaMKII α, CaMKII β, CaMKII δ, CaMKII γ, and GAPDH. These primer sets were synthesized at IDT (PrimeTime qPCR primer assays). GAPDH was used as a reference gene. The 2^−∆∆C^_T_ method was used to calculate fold changes in gene expression. All experiments were repeated at least three times.

### 2.6. Immunocytochemistry

U251 cells growing on coverslips were incubated with KN-93 (10 μM); they were then transfected with CaMKIIβ siRNA to silence the expression of CaMKIIβ and incubated for an additional 24 h. Cells were fixed in 4% paraformaldehyde (PFA) for 20 min at room temperature (about 20–25 °C) and then incubated with Wheat Germ Agglutinin, Alexa Fluor^®^ 647 conjugate (WGA647) (1:200; Thermo Fisher Scientific, W11262) at 4 °C for 15 min to label the plasma membrane. Cells were then permeabilized with Triton X-100 (0.5% in PBS) and blocked with 5% bovine serum albumin for 1 h at room temperature. Subsequently, the cells were incubated overnight at 4 °C with anti-ANO1 (1:200, Santacruz, sc377115) antibody; after washing, cells were incubated with DyLight 488-conjugated secondary antibody (1:500, Invitrogen, Carlsbad, CA, USA, A21202) for 1 h at room temperature. The cells were then washed three times, mounted after drying, and observed under a Nikon A1 confocal microscope.

### 2.7. Surface Biotinylation Assay

For the surface biotinylation assay, U251 cells transfected with CaMKIIβ siRNA or CaMKII isoforms were incubated at 4 °C and washed with PBS twice. Plasma membrane proteins were then biotinylated in PBS containing sulfo-NHS-LS-biotin (Thermo Fisher Scientific, 1335) for 30 min. After biotinylation, cells were washed with quenching buffer (100 mM glycine in PBS) and then washed with PBS two times. Cells were then lysed and incubated with high-capacity NeutrAvidin-agarose resin (Thermo Fisher Scientific, 29204). After three washes with lysis buffer, bound proteins were eluted in SDS sample buffer and separated using 10% SDS PAGE electrophoresis; they were then transferred to PVDF membranes. After blocking, membranes were incubated overnight at 4 °C with anti-ANO1 (LS-Bio, Seattle, USA, C405940), anti-Flag (M-2, Sigma), or anti CaMKIIβ (Thermo Fisher Scientific, PA5-29327) antibody and were then incubated with HRP-conjugated anti-mouse, anti-rat, or anti-rabbit IgG antibody.

### 2.8. Electrophysiological Recording for Cell

Current-voltage (*I–V*) curves were obtained using U251 cells transfected with mCherry-CaMKII isoforms, and whole-cell currents were measured by applying 1-s duration voltage ramps from +100 to −100 mV (a holding potential of −10 mV) at room temperature. For measuring Cl^−^ currents, recording electrodes (4–7 MΩ) were filled with (mM): 146 CsCl, 8 HEPES, 5 Ca-EGTA-NMDG, 2 MgCl_2_, and 10 sucrose (pH adjusted to 7.3 with CsOH). The standard bath solution contained (in mM) 139 NaCl, 3 KCl, 10 HEPES, 2 MgCl_2_, 2 CaCl_2_, and 5.5 glucose (pH adjusted to 7.3 with NaOH). Whole-cell currents were amplified using the Axopatch 200A patch clamp system. Acquired data were analyzed using the pCLAMP 10.2 software (Molecular Devices). 

### 2.9. Migration Assay

Glioblastoma cells were infected or transfected with ANO1 shRNA lentivirus or CaMKIIβ siRNA. These cells were plated onto SPLScar™ Scratcher culture dish (SPL Life Sciences, Pocheon, Korea, 201935) at a density of 1 × 10^5^ cells per well. After 24 h, SPLScar™ blocks in culture dish were removed from transfected cells to artificially generate cell free gap of 500 μm thick wall insuring higher uniformity and reproducibility. These cells were incubated in complete media for 16 h (U251 cells) or 24 h (U87MG cells). Phase contrast images were captured using ECLIPSE Ts2 inverted Routine Microscope (Nikon, Tokyo, Japan). Cancer cells were rapidly grown in a small space of SPLScar™ Scratcher culture dish, phase of the growing cells can be different from that of initially seeding cells. Analyses were performed using image J which select outlines where the wound is partially closed. The ratio of the remaining wound area was calculated relative to the initial wound area and normalized to that for scrambled shRNA-infected cells and scrambled siRNA-transfected cells. The error bars in graphs denote the standard error of the mean (s.e.m). All experiments were performed in triplicate.

### 2.10. Cell Invasion Assay

Trans-well invasion chambers with 8.0 mm pores (Corning, New York, USA, 353097) were used, according to the manufacturer’s instructions. Growth factor reduced Matrigel (Corning, 354230) was used to coat the membrane for 5 h. U251 cells or U87 MG cells infected with ANO1 shRNA lentivirus or transfected with CaMKIIβ siRNA were seeded onto the transwell membrane insert at a density of 1 × 10^5^ cells/well in 100 μL DMEM. The lower chambers were filled with 500 μL DMEM. The trans-wells were incubated for 18 h to allow cell migration. Following incubation, the cells from the upper side of the insert filter were completely removed using a cotton swab, and those that had invaded through the coated membrane to its lower surface were fixed with 100% ethanol and stained with hematoxylin and eosin. For quantification, cells were counted under a microscope in three random fields at 20× magnification. All experiments were performed in triplicate.

### 2.11. Cell Proliferation Assay

U251 cells were seeded in 96-well plates at a density of 5 × 10^3^ cells/well and incubated for 24 h. Cells were infected or transfected with ANO1 shRNA lentivirus or CaMKIIβ siRNA. After 48 h, the reagents of 3-(4,5-dimethylthiazol-2-yl)-2,5-diphenyl tetrazolium bromide (MTT) was made with D-Plus™ cell counting kit (CCK) cell viability assay kit (Dong-in LS, Seoul, Korea, CCK-3000) according to the manufacturer’s protocol. These reagents were added to each well and the cells were incubated for 1 h at 37 °C. After incubation, Dimethyl sulfoxide (DMSO) is added at the end of the reaction after the medium from cells were removed to dissolve the formazan crystals formed from the reaction. After 30 min, the absorbance was measured at 450 nm wavelength using a spectrophotometer (Molecular Devices, Mountain View, CA, USA).

### 2.12. Statistical Analysis

Statistical analysis was performed using Origin software (ver7.0, Origin Lab cooperation). The sample size employed was based on previous studies and was not predetermined by a statistical method. No randomization method was used. Data distribution was assumed to be normal, but this was not formally tested. Numerical data are presented as mean ± standard error of the mean (s.e.m). The variances were similar between the groups compared. Statistical significance was assessed using an unpaired or paired Student’s t-test, with the significance level denoted by asterisks (* *p* < 0.05, ** *p* < 0.01, or *** *p* < 0.001).

## 3. Results

### 3.1. KN-93, a Selective CaMKII Blocker, Reduces Migration and Chloride Currents in U251 Cells

**Figure 1:**
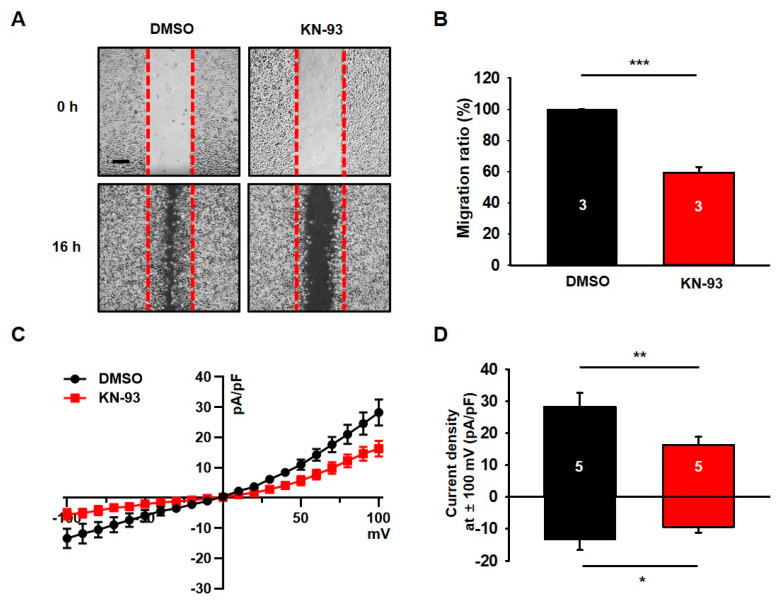
The CaMKII blocker KN-93 decreases the migration and chloride currents of U251 cells. (**A**) Representative photographic images showing the migration of U251 cells treated with KN-93 as compared with the migration of the cells treated with dimethyl sulfoxide (DMSO). Scale bar, 200 μm. (**B**) The summary bar graph shows the reduced migration of U251 cells after treatment with KN-93 compared to that of cells treated with DMSO. (**C**) Averaged traces of whole-cell currents of U251 cells treated with KN-93. (**D**) The summary bar graph shows the inhibitory effect of KN-93 on the amplitude of the chloride current at ± 100 mV. Number on each bar indicates n for each condition. All values are mean ± s.e.m. *P*-values were obtained with Student’s t-test. * *p* < 0.05, ** *p* < 0.01, and *** *p* < 0.001.

Since KN-93, a CaMKII blocker, inhibited cell growth and neurosphere formation in U87 MG cells [32], it is plausible that KN-93 also suppresses the cell growth in other glioblastoma cell lines. To test this possibility, we examined the effect of KN-93 on the tumorigenesis of U251 glioblastoma cells. As shown in *A and B, we found that the treatment of KN-93 clearly decreased about 40% of the migration capability in U251 cells. Based on previous studies showing that chloride channels are involved in the migration of cancer cells [10,33], we next examined whether channel activity of chloride channels can be altered by KN-93 in U251 cells. Chloride currents were measured by whole-cell configuration of patch-clamp recording with symmetrical chloride solutions. The current-voltage (*I–V*) relationship of chloride currents in U251 cells showed outward and inward currents (Figure 1C,D). In the presence of 10 μM KN-93, chloride currents are inhibited by about 40% of control currents in U251 cells (Figure 1C,D). In addition, in the presence of DIDS, a chloride channel blocker, sensitive chloride currents were almost completely suppressed by the KN-93 (data not shown).

These results clearly indicate that CaMKII is involved in the regulation mechanism of chloride channels and the cellular process involved in migration in U251 glioblastoma cells.

### 3.2. KN-93 Reduces the Surface Expression and Activity of ANO1 in U251 Cells

**Figure 2:**
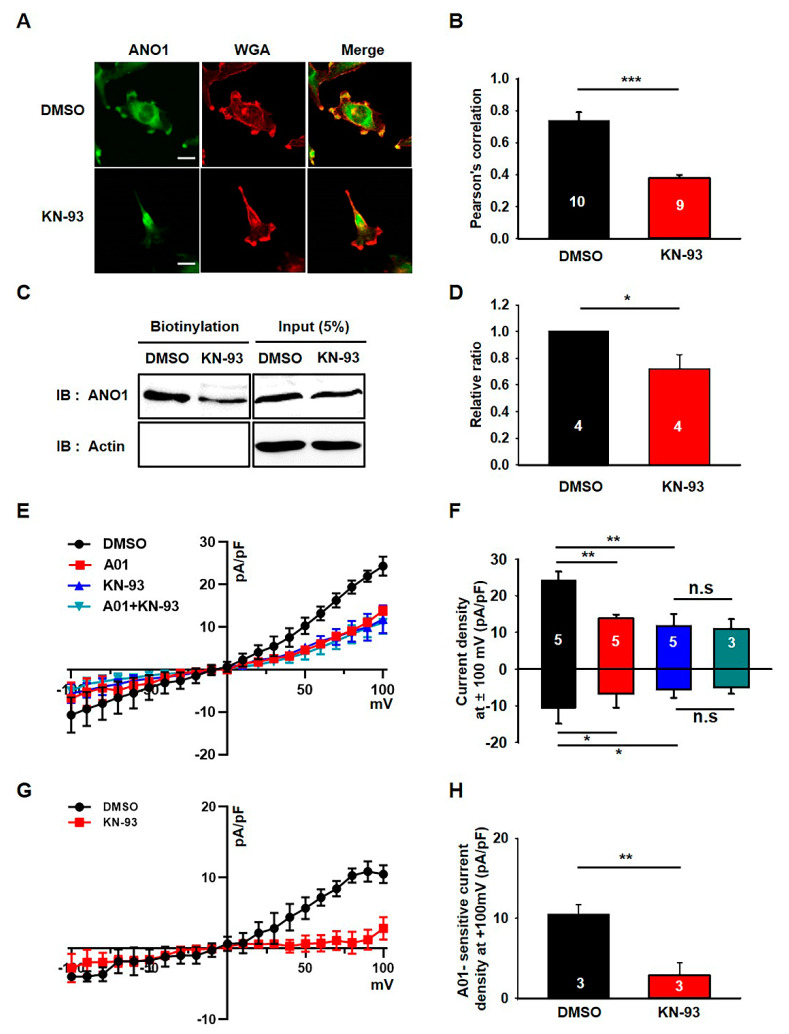
KN-93 reduces the surface expression and activity of ANO1 in U251 cells. (**A**) U251 cells treated with DMSO or KN-93 were imaged using antibodies against ANO1 and WGA647 (WGA), a plasma membrane marker. Scale bar, 20 μm. (**B**) The Pearson’s correlation coefficient for ANO1 with KN-93 was significantly less than the value obtained for ANO1 with DMSO in U251 cells. (**C**) Cell surface biotinylation results from membrane protein fractions from U251 cells treated with DMSO or KN-93. (**D**) The summary bar graph showing data obtained from three independent experiments as in (**C**). (**E**) Averaged traces of whole-cell currents of U251 cells treated with DMSO or T16Ainh-A01, an ANO1 inhibitor. (**F**) The summary bar graph shows the inhibitory effect of KN93 or T16Ainh-A01 on ANO1 current amplitude at ±100 mV. (**G**) Averaged traces of normalized T16Ainh-A01-sensitive currents of U251 cells treated with DMSO or KN-93. (**H**) The bar graph shows normalized T16Ainh-A01-sensitive current densities (**G**) at + 100 mV. Number on each bar indicates n for each condition. All values are mean ± s.e.m. *P*-values were obtained with Student’s t-test. * *p* < 0.05, ** *p* < 0.01, and *** *p* < 0.001. n.s means not significant.

We previously demonstrated that the ANO1 chloride channel was highly expressed in U251 cells and that its surface expression was critical for their migration [10]. Therefore, it seems that the ANO1 channel may be a primary target for the effects of KN-93 in these cells. To confirm this possibility, we next examined the effect of KN-93 on the surface expression and channel activity of ANO1 in U251 cells. Immunocytochemical data showed that treatment with KN-93 led to a prominent reduction in ANO1 localization at the plasma membrane of U251 cells (t-test; *p* = 0.0008) (Figure 2A,B). ANO1 and WGA647, a fluorescent-labeled wheat germ agglutinin labeling membrane glycoprotein (or glycolipid), are rarely co-localized in U251 cells under the treatment of KN-93, whereas ANO1 is clearly co-localized with WGA647 at the plasma membrane of naïve U251 cells. The comparison of Pearson’s correlation coefficients showed that ANO1 expression at the plasma membrane was significantly reduced by treatment with KN-93. In addition, the surface biotinylation assay also confirmed that KN-93 treatment caused a significant reduction in ANO1 surface expression without affecting the total ANO1 protein levels in U251 cells (t-test; *p* = 0.014) (Figure 2C,D). We also found that the chloride currents of U251 cells were prominently inhibited by treatment by KN-93 or T16A_inh_-A01, an ANO1-specific inhibitor (Figure 2E,F). Figure 2G,H shows that the A01- sensitive chloride current was almost completely inhibited by KN-93. These data demonstrated that the surface expression and channel activity of ANO1 were reduced by KN-93, a selective CaMKII inhibitor, in U251 glioblastoma cells.

### 3.3. CaMKIIβ Specifically Increases the Surface Expression and Activity of ANO1 in U251 Cells

**Figure 3:**
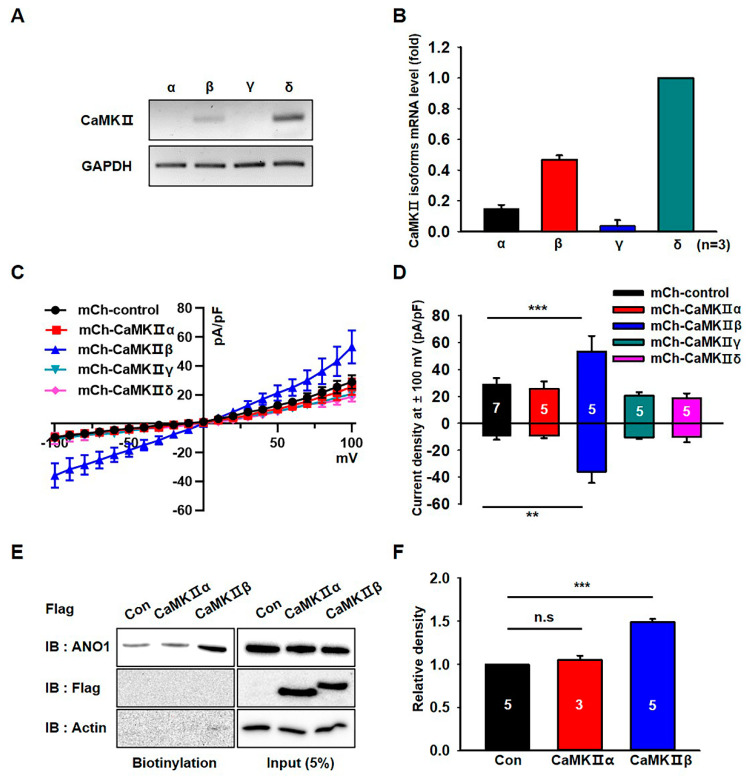
CaMKIIβ specifically increases the surface expression and activity of ANO1 in U251 cells. (**A**) The mRNA expression of CaMKII isoforms in U251 cells were detected with specific primers for CaMKIIα, β, γ and δ. (**B**) Normalized expression of CaMKII isoforms as compared to that of GAPDH in U251 cells. (**C**) Effect of CaMKII isoform overexpression on ANO1-mediated whole cell chloride currents in U251 cells. (**D**) The summary bar graph showing ANO1-mediated current density at ± 100 mV. (**E**) Cell surface biotinylation results from membrane protein fractions from U251 cells transfected with mCh-CaMKIIα and mCh-CaMKIIβ. (**F**) The summary bar graph showing data obtained from at least three independent experiments as in (**E**). Number on each bar indicates n for each condition. All values are mean ± s.e.m. *P*-values were obtained with Student’s t-test. ** *p* < 0.01 and *** *p* < 0.001. n.s means not significant.

We next analyzed the expression of CaMKII isoforms in U251 cells. RT-PCR experiments demonstrated that the mRNA levels of CaMKIIβ and CaMKIIδ were higher than those of CaMKIIα and CaMKIIγ in U251 cells (Figure 3A,B). To identify which CaMKII isoforms are critical for the channel activity of ANO1, we examined the chloride currents in U251 cells transfected with CaMKII isoforms. As shown in Figure 3C,D, endogenous chloride currents were specifically increased by mCherry-tagged CaMKIIβ (mCh- CaMKIIβ) compared to those of other isoforms. Although CaMKIIδ was the most highly expressed isoform of the CaMKII family in U251 cells, only CaMKIIβ was shown to affect the increase of chloride currents in U251 cells. In addition, the surface biotinylation assay showed that only CaMKIIβ overexpression significantly increased the surface expression of ANO1 in U251 cells (t-test; *p* = 0.0005) (Figure 3E,F and Figure S1). These data clearly demonstrated that only CaMKIIβ among the CaMKII family members was involved in the surface expression and channel activity of ANO1 in U251 glioblastoma cells.

### 3.4. CaMKIIβ Knockdown Reduces the Surface Expression of ANO1 in U251 Cells

**Figure 4:**
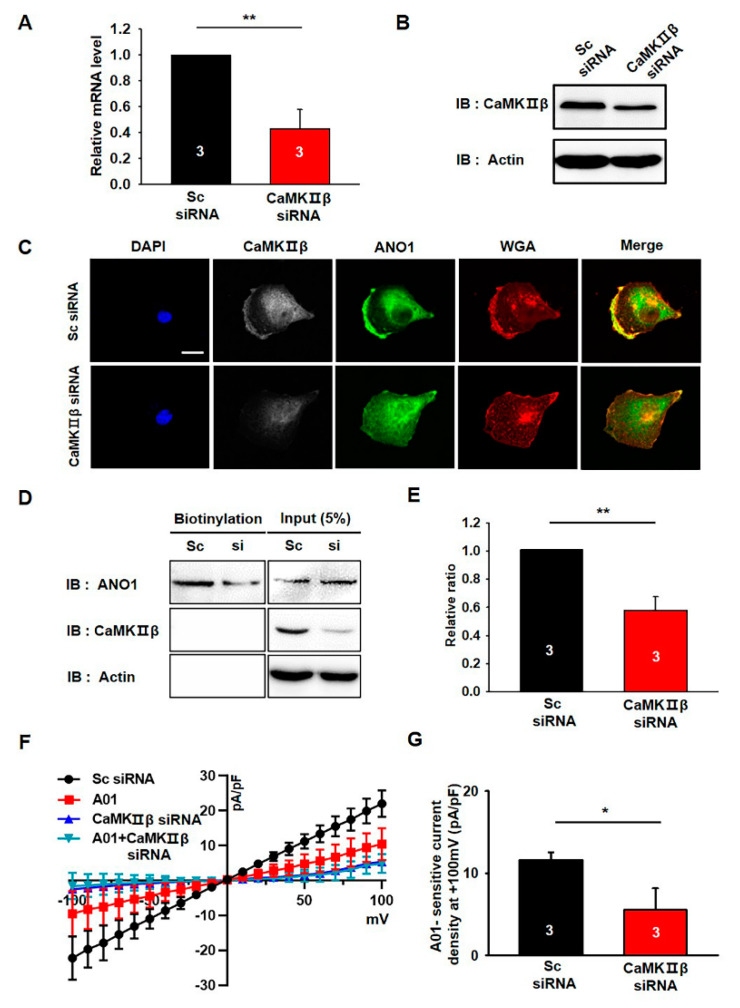
CaMKIIβ knockdown reduces the surface expression of ANO1 in U251 cells. (**A,B**) Validation of the silencing efficiency of the siRNA against CaMKIIβ using qPCR and Western blotting. (**C**) U251 cells transfected with Sc shRNA or CaMKIIβ siRNA were imaged using antibodies against ANO1 and WGA. Nuclei were stained using DAPI staining solution. Scale bar, 20 μm. (**D**) Cell surface biotinylation results from U251 cells transfected with Sc shRNA or CaMKIIβ siRNA. (**E**) The summary bar graph shows the summary of (**D**), data obtained from three independent experiments. (**F**) Averaged traces of whole-cell currents of U251 cells transfected with Sc shRNA, CaMKIIβ siRNA, or T16Ainh-A01 (A01). (**G**) The summary bar graph shows the inhibitory effect of CaMKIIβ siRNA on ANO1–mediated current amplitude at ±100 mV. The bar graph shows normalized A01-sensitive current densities at + 100 mV. Number on each bar indicates n for each condition. All values are mean ± s.e.m. *p*-values were obtained with Student’s t-test. * *p* < 0.05 and ** *p* < 0.01.

We examined the effects of CaMKIIβ knockdown on the surface expression and channel activity of endogenous ANO1 in U251 cells using CaMKIIβ siRNA. The knockdown efficiency of CaMKIIβ siRNA was examined by qPCR and Western blotting experiments (Figure 4A,B). The mRNA and protein levels of endogenous CaMKIIβ were clearly reduced by approximately 60% following treatment with CaMKIIβ siRNA.

Since ANO1 surface expression is specifically increased by CaMKIIβ overexpression in the heterologous expression system (Figure 3), we next examined the effect of CaMKIIβ siRNA on the surface expression of endogenous ANO1 in U251 cells. As shown in Figure 4C, ANO1 is highly co-localized with WGA647 at the plasma membrane of U251 cells transfected with scrambled siRNA (Sc siRNA), whereas ANO1 is less co-localized with WGA647 in CaMKIIβ siRNA-transfected U251 cells. The surface biotinylation assay also showed that knockdown of CaMKIIβ caused a significant reduction in ANO1 surface expression without affecting the total protein levels of ANO1 (t-test; *p* = 0.009) (Figure 4D,E). In addition, electrophysiolgical data clearly showed that knockdown of CaMKIIβ with siRNA inhibited ANO1-mediated chloride currents in U251 cells (Figure 4F,G).

These data clearly demonstrated that gene silencing of CaMKIIβ reduces the surface expression of ANO1, as well as ANO1-mediated chloride currents in U251 glioblastoma cells.

### 3.5. Deficiency of ANO1 or CaMKIIβ Suppresses Invasion and Migration of Glioblastoma Cells

**Figure 5:**
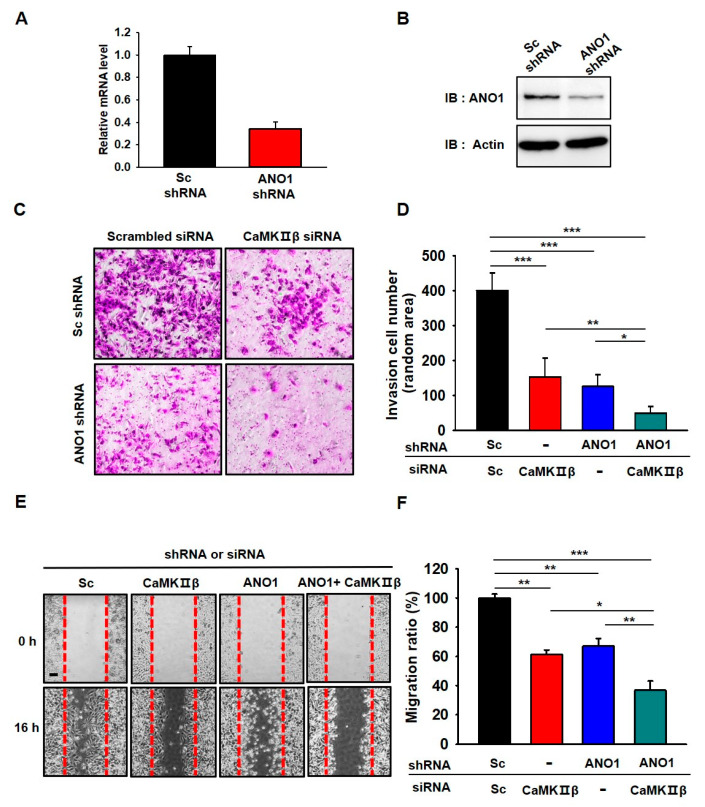
Gene silencing of ANO1 or CaMKIIβ leads to the attenuation of migration and invasion in U251 cells. (**A,B**) Validation of the silencing efficiency of the shRNA against ANO1 using qPCR and Western blotting. (**C**) Cell invasion assay of U251 cells infected with Lenti-ANO1 shRNA and transfected with CaMKIIβ siRNA. (**D**) The averaged bar graph shows the summary of (**C**), data obtained from three independent experiments. (**E**) Representative photographic images of the migration of U251 cells infected with Lenti-ANO1 shRNA and transfected with CaMKIIβ siRNA and the cells with Lenti-Sc shRNA and Sc siRNA control. Scale bar, 100 μm. (**F**) The averaged bar graph shows the summary of (**E**), data obtained from three independent experiments. All values are mean ± s.e.m. *p*-values were obtained with Student’s t-test. * *p* < 0.05, ** *p* < 0.01, and *** *p* < 0.001.

We recently reported that surface expression and channel activity of ANO1 are critical in the tumorigenesis of U251 cells [10]. Since ANO1 surface expression and chloride currents were regulated by CaMKIIβ in U251 cells, we examined whether gene silencing of CaMKIIβ or ANO1 affect the characteristics of cancer cells, such as invasion and migration. First, we examined the knockdown efficiency of ANO1 shRNA, which was examined by qPCR and Western blotting experiments (Figure 5A,B). The mRNA and protein levels of endogenous ANO1 in U251 glioblastoma cells were clearly reduced by approximately 70% following treatment with ANO1 shRNA. 

As expected, depletion of ANO1 or CaMKIIβ in U251 cells resulted in a significant decrease in the invasiveness (as assessed by the collagen-coated transwell invasion assay) of U251 cells compared to the invasiveness of Sc shRNA-and Sc siRNA-treated cells (Figure 5C,D). In addition, the invasiveness of these cells was additively suppressed by co-expression of both ANO1 shRNA and CaMKIIβ siRNA. We also examined the silencing effect of ANO1 shRNA and CaMKIIβ siRNA on migration (via the wound-healing assay) in U251 cells (Figure 5E,F). The wound area recovered rapidly (within 16 h) in Sc shRN-A- and Sc siRNA-treated cells. However, wound closure was delayed in ANO1 shRNA- and CaMKIIβ siRNA-treated cells when examined at the same time point. There was an additive effect in the wound healing assay when both ANO1 shRNA and CaMKIIβ siRNA were used as treatments. We also evaluated the silencing effect of ANO1 shRNA and CaMKIIβ siRNA on proliferation (via the MTT assay) in the cells (Figure S2). The results showed that the downregulation of ANO1 or CaMKIIβ expression resulted in a decrease in proliferation of U251 cells compared to the Sc shRNA-and Sc siRNA-treated cells. The proliferation of U251 cells was additively suppressed by co-expression of both ANO1 shRNA and CaMKIIβ siRNA.

**Figure 6:**
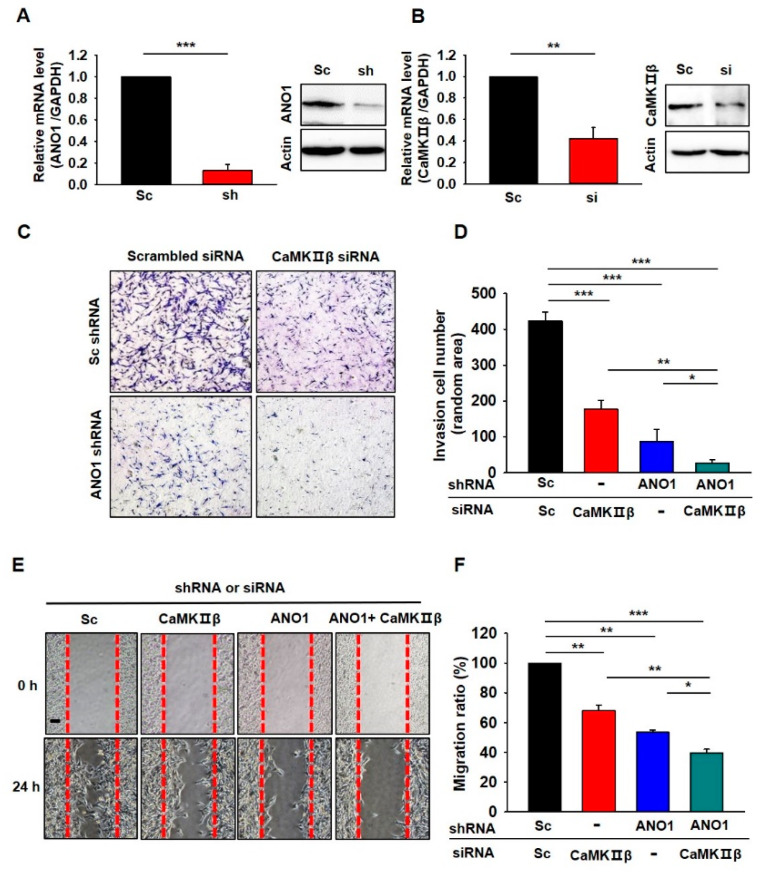
Inhibition of CaMKIIβ and/or ANO1 decreased migration and invasion of U87MG cells. (**A,B**) Validation of the silencing efficiency of the Lenti-ANO1shRNA and the CaMKIIβ siRNA with qPCR and Western blot in U87MG cells. (**C**) Cell invasion assay of U87MG cells infected with Lenti-ANO1shRNA and transfected with CaMKIIβ siRNA. (**D**) The averaged bar graph shows the summary of (C), data obtained from three independent experiments. (**E**) Representative photographic images of the migration of U87MG cells infected with Lenti-ANO1shRNA and transfected with CaMKIIβ siRNA as compared to the cells with Lenti-Sc shRNA and Sc siRNA control. Scale bar, 100 μm. (**F**) The averaged bar graph shows the summary of (**E**), data obtained from three independent experiments. All values are mean ± s.e.m. *p*-values were obtained with Student’s t-test. * *p* < 0.05, ** *p* < 0.01, and *** *p* < 0.001.

Finally, we examined whether the deficiency of CaMKIIβ or ANO1 also affects other glioblastoma cells. We tested the roles of CaMKIIβ and ANO1 in the tumorigenesis of U87 MG cells which is another glioblastoma cell showing high expression of ANO1 [22]. As shown in Figure 6A,B, both ANO1 shRNA and CaMKIIβ siRNA successfully suppressed the mRNA and protein levels of their targets. The invasion assay and wound-healing assay in U87 MG cells also demonstrated that depletion of ANO1 or/and CaMKIIβ exhibited similar effects (Figure 6).

Cumulatively, these results strongly suggest that suppression of ANO1 surface expression by knockdown of CaMKIIβ is critical in the progression of human glioblastoma.

## 4. Discussion

We previously reported that the surface expression and channel activity of ANO1 are critical for the tumorigenesis of glioblastoma cells [10]. However, regulation mechanisms of ANO1 surface expression are largely unknown. The present study uncovers the pivotal role of CaMKIIβ in the surface expression of ANO1 at the plasma membrane of glioblastoma cells. We have found evidence demonstrating that CaMKIIβ specifically increases the surface expression and channel activity of ANO1 in a heterologous expression system. In addition, we have shown that knockdown of CaMKIIβ reduces the surface expression and channel activity of ANO1 in U251 glioblastoma cells, demonstrating the oncogenic roles of CaMKIIβ-mediated ANO1 surface expression in U251 and U87 MG glioblastoma cells. 

CaMKII has been identified as a factor in the proliferation, migration, and survival of various cancer cells, such as those of lung, breast, prostate, and colon cancers [20,34,35,36]. Similar to the important roles of CaMKII in other cancers, several studies have shown that CaMKII plays a critical role in the migration and invasion of glioma cells. In D54 glioma cells, CaMKII enhanced migration of glioma cells via an increase in ClC-3 currents [37]. A more recent study showed cell growth and neurosphere formation of U87 MG globlastoma cells were suppressed by treatment with KN-93, an inhibitor of CaMKII, or the knockdown of CaMKIIγ [32]. Consistent with these results, our study clearly showed that KN-93 reduces surface expression and channel activity of ANO1, and migration of U251 glioblastoma cells (Figure 1 and Figure 2). Interestingly, we noted that in U251 glioblastoma cells, CaMKIIβ and not CaMKIIγ are involved in the increase of ANO1 surface expression and ANO1 channel activity (Figure 3, Figure 4 and Figure S1). In addition, knockdown of CaMKIIβ significantly suppressed the migration, invasion, and proliferation of U251 cells (Figure 5, Figure 6, and Figure S2). These studies strongly suggest that CaMKII isoforms seem to be involved in the tumorigenesis of glioblastoma cells via the regulation of chloride channels, raising the possibility that a different set of CaMKII isoforms and corresponding chloride channels may be working in different glioblastoma cells. 

It is noteworthy that previous studies demonstrated functional interactions between CaMKII and ANO1 chloride channels [20,29,30]. In breast cancer cells, ANO1 promotes cancer progression by stimulating the cell proliferation signaling pathway involving EGFR and CaMKII [20]. The knockdown of ANO1 or pharmacological inhibition of ANO1 activity has been shown to clearly reduce EGFR and CaMKII signaling [20]. On the contrary, CaMKII can decrease ANO1 activity in basilar arterial smooth muscle cells [29] and HEK293 cells expressing mouse ANO1 [30]. These studies suggest the existence of crosstalk between CaMKII, a well-known calcium-activated kinase, and ANO1, a calcium-activated chloride channel in both normal and cancer cells. In line with these studies, we also found that the pharmacological inhibition of CaMKII with KN-93 or the specific knockdown of CaMKIIβ suppressed the surface expression and channel activity of ANO1 in U251 glioblastoma cells (Figure 2 and Figure 4). In general, intracellular Ca^2+^ concentration is critical for the cancerous progression of glioblastoma cells [9,38,39,40]. Since ANO1 is activated by intracellular Ca^2+^ [12,13,14], it is plausible that diverse receptor-mediated increases in intracellular Ca^2+^ can enhance surface expression and channel activity of ANO1 via activation of CaMKIIβ. The relationship between receptor-mediated Ca^2+^ signaling and ANO1-mediated tumorigenesis should be investigated in a future study. 

We previously reported that the surface expression and channel activity of ANO1 can be regulated by ANO1-interacting proteins, such as 14-3-3γ and β-COP, in glioblastoma cells [10,11]. In addition, a recent study also showed that CaMKIIγ, a novel ANO1-interacting protein, inhibits ANO1-currents in BASMC [29]. Since a large number of ANO1-interacting proteins were discovered using proteomics techniques in ANO1 over-expressing HEKT293 cells and the HNSCC cell line Tel1 [20,41], it is plausible that these putative ANO1-interacting proteins may also be involved in the regulatory mechanisms of ANO1-mediated tumorigenesis in ANO1-enriched cancer cells, including glioblastoma cells. Therefore, these putative interacting proteins for ANO1 are useful for understanding the regulatory network of ANO1 in glioblastoma cells, although the detailed functions of each interaction should be examined in the ANO1-mediated cancer progression of glioblastoma cells. Because most ion channels act at the plasma membrane, clarifying the trafficking mechanisms of ANO1 channels at the plasma membrane is important for developing potent therapeutic approaches for glioblastomas. In addition, understanding the molecular mechanisms of ANO1 trafficking will help expand our knowledge of the physiological roles of ANO1 in various tissues and in other ANO1-related diseases.

Our results showed that CaMKIIβ and CaMKIIδ were expressed in U251 glioblastoma cells and only CaMKIIβ-overexpression enhanced the ANO1-mediated current in the cells (Figure 2). However, recent studies reported that ANO1 currents were inhibited by CaMKII*γ* phosphorylation at serine 727 of ANO1 in cerebrovascular cells [29] and CaMKII phosphorylation at serine 525 of ANO1 in HEK293 cells expressing mouse ANO1 [30]. These two serine residues of ANO1 were predicted by Group-based Prediction System software (http://gps.biocuckoo.org) and confirmed the possible CaMKII phosphorylation site(s) by site-directed mutagenesis experiments tested in these studies. Because of the different cell types and experimental conditions, it seems that they provided different serine residues of ANO1 as CaMKII-dependent phosphorylation sites. However, we can exclude the involvement of CaMKIIγ in the inhibitory effect of KN-93 on ANO1 currents and the migration of U251 cells, as CaMKIIγ was not expressed in U251 glioblastoma cells (Figure 3). Unfortunately, when serine residues at 525 and 727 of ANO1 were mutated to a neutral alanine by site-directed mutagenesis, these mutations were not found to influence the effect of CaMKIIβ on ANO1 currents in U251 cells (data not shown). These results suggested that other residue(s) of ANO1 are required for CaMKIIβ-targeted phosphorylation site(s) in glioblastoma cells. Therefore, the CaMKIIβ-mediated phosphorylation site(s) of ANO1 should be examined in a future study. 

In conclusion, the CaMKIIβ-mediated regulation of ANO1 surface expression plays a critical role in the oncogenic properties of glioblastoma cells. The specific regulatory mechanism of ANO1 by CaMKIIβ should prove to be helpful in understanding the functional roles of ANO1 in glioblastoma cells. Eventually, this finding may advance research into efficient therapeutic targets for various ANO1-mediated cancers, including glioblastoma.

## Figures and Tables

**Table 1:** Primer sets for PCR and qPCR.

Gene		Sequence	Size
CaMKIIα	Forward Primer Reverse Primer	5′- AGGCTTCAATTCCCAGCTCT -3′ 5′- TGTGTCAGCCAATGAAAGGC -3′	137
CaMKIIβ	Forward Primer Reverse Primer	5′- CCAGAAGCTGGAGAGAGAGG-3′ 5′- AAGACCAGGTAGTGGAAGCC -3′	105
CaMKIIδ	Forward Primer Reverse Primer	5′- GATTTCTGCTGACAGTGCGT-3′ 5′- GAGTAGGACACACTGCCCTT-3′	100
CaMKIIγ	Forward Primer Reverse Primer	5′-CTTCTCTCACAGGAGCCACA-3′ 5′-TCTCCTGCTGACCTGGAAAG-3′	104
GAPDH	Forward Primer Reverse Primer	5′-CCATGGAGAAGGCTGG-3′ 5′-CAAAGTTGTCAGGATGACC-3′	195

## Supplementary Materials

The following are available online at https://www.mdpi.com/2073-4409/9/5/1079/s1. Figure S1: The surface expression and activity of ANO1 is not affected by CaMKIIδ and CaMKIIγ co expression in U251 cells. Figure S2: Silencing of CaMKⅡβ and/or ANO1 reduced proliferation of U251 cells.
