# Supplementary material for: Suppression of CaMKIIβ Inhibits ANO1-Mediated Glioblastoma Progression
Source: Cells. 2020 Apr 26;9(5):1079. doi: 10.3390/cells9051079 (PMC7290681; doi:10.3390/cells9051079)
Supplement: Supplementary file 1 [file cells-09-01079-s001.pdf]

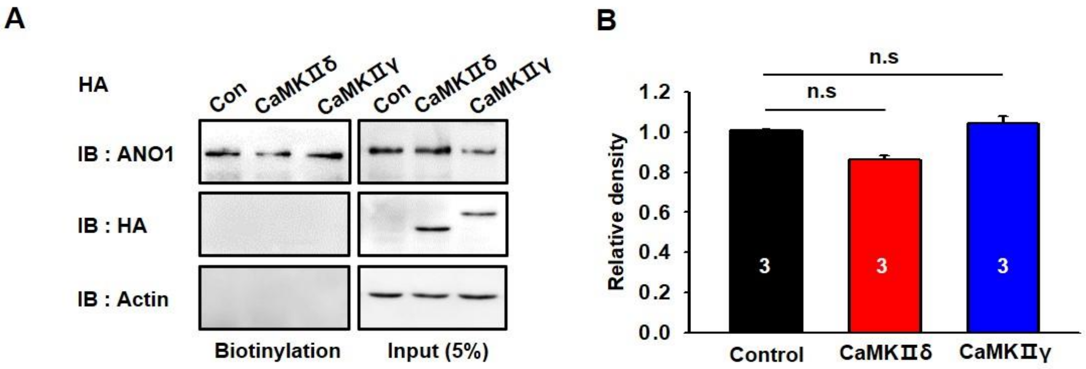

Supplemental Figure 1. The surface expression and activity of ANO1 is not affected by CaMKIIδ and CaMKIIγ co-expression in U251 cells. (**A**) Cell surface biotinylation results from membrane protein fractions from U251 cells transfected with CaMKIIδ or CaMKIIγ. (**B**) A normalized bar graph shows the summary of (**A**), data obtained from three independent experiments.

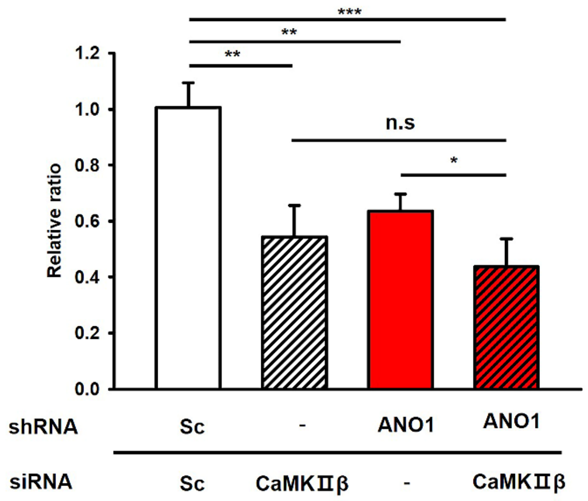

Supplemental Figure 2. Silencing of CaMKIIβ and/or ANO1 reduced proliferation of U251 cells**.** Proliferation assays were performed with U251 cells infected with Lenti-ANO1shRNA and/or transfected with CaMKIIβ siRNA. Normalized bar graphs showing data obtained from three independent experiments. Silencing effects of CaMKIIβ or ANO1 on proliferation were compared to proliferation of the control cells treated with Lenti-Sc shRNA and Sc siRNA.
